# Identifying high-risk population segments for underweight, overweight, and obesity among reproductive-age women in sub-Saharan Africa

**DOI:** 10.3389/fpubh.2024.1467747

**Published:** 2025-01-23

**Authors:** Amare Abera Tareke, Anissa Mohammed, Amare Muche, Yeshimebet Ali

**Affiliations:** ^1^Department of Biomedical Sciences, School of Medicine, Wollo University, Dessie, Ethiopia; ^2^Department of Epidemiology and Biostatistics, School of Public Health, Wollo University, Dessie, Ethiopia; ^3^Department of Nutrition, School of Public Health, Wollo University, Dessie, Ethiopia

**Keywords:** underweight, overweight, obesity, women, sub-Saharan Africa, prevalence

## Abstract

**Background:**

Despite significant progress in addressing underweight in developing countries, the recent rise in the number of overweight and obese individuals has confirmed that the double burden of malnutrition will remain a crucial problem in the foreseeable future. Some countries that previously succeeded in reducing underweight rates are now experiencing a resurgence. Initiatives in sub-Saharan African (SSA) countries aimed at reducing malnutrition often lack robust evidence. This study aimed to identify risk groups for malnutrition among women of reproductive age in SSA countries and prioritize intervention areas.

**Methods:**

This analysis utilized data from 247,911 reproductive-age women across recent demographic and health surveys conducted in 33 SSA countries. Nutritional status was assessed using body mass index (BMI). We computed the pooled prevalence of different forms of malnutrition using the random effects inverse variance method. We evaluated the factors associated with different forms of malnutrition using multilevel multinomial regression. We reported the adjusted odds ratios and 95% confidence intervals (CIs).

**Results:**

The pooled prevalence of underweight, overweight, and obesity among SSA women was 11% (95% CI: 9–12%), 18% (95% CI: 16–20%), and 10% (95% CI: 8–12%), respectively. Significant factors influencing malnutrition included women’s age, highest educational level, wealth index, current breastfeeding status, contraceptive use, parity, media exposure, marital status, place of residence, and regional location within SSA. Factors such as education, wealth, age, contraceptive use, parity, and marital status were risk factors for overweight and obesity but were protective against underweight. Employment was protective against all three malnutrition forms.

**Conclusion:**

Increased age, wealth index, not-breastfeeding status during the survey, contraceptive use, higher parity, marital status, urban residency, and living in southern or central Africa are associated with higher odds of increased BMI and lower odds of underweight. In designing interventions for overweight and obesity, emphasis should be given to the wealthy, reproductive-age women in later age, urban residents, and multiparous. Whereas the poorest and youngest is priority intervention segments for underweight.

## Introduction

While significant progress has been made in developing countries in addressing underweight, the recent development of overweight and obesity has affirmed that the double burden of malnutrition will remain a crucial problem in the foreseeable future (1). Globally, the increasing mean body mass index (BMI) has significantly raised the burden of diseases linked to high BMI (2, 3). Accompanying the increase in mean BMI, the prevalence of overweight and obesity is rising at an alarming rate in many parts of the world. In 2022, the World Health Organization (WHO) reported that 2.5 billion adults aged 18 years and older were overweight, and 890 million adults were obese (4); this translates to 43 and 16% of the world’s adult population being overweight and obese, respectively (4). In Africa, the prevalence of overweight and obesity either doubled or tripled in half of the 24 countries in urban areas (5). The pooled prevalence of underweight, overweight, and obesity from Demographic and Health Surveys (DHS) of 32 African countries was 8.87, 16.47, and 6.10%, respectively, among reproductive-age women (6). The prevalence of undernourishment in the general population globally was 19.1% in 2019, and this figure is expected to increase to 25.7% by 2030 (7).

While undernutrition is known to affect developing countries, including SSA, the burden of overweight and obesity is no longer concentrated in high-income countries and affluent communities of developing countries (8). The prevalence and mortality are rising at an alarming rate among LMICs and the lowest decile of the community in the wealth index due to the rapid nutrition transition (9, 10).

From 2000 to 2019, the age-specific death rate (ASDR) in Africa increased annually by 0.86%, while in Europe and America, it decreased annually by −1.16% and −0.27%, respectively (9). This stark contrast highlights the severity of the health challenges faced by Africa. Additionally, regions such as SSA grapple with the dual burden of undernutrition and the rising prevalence of overweight and obesity (11, 12).

Several studies evaluated the factors associated with underweight, overweight, and obesity. For example, older age, being married, higher economic status, being employed, urban residence, and alcohol use were associated with overweight and obesity in Zimbabwean women (13). Whereas, maternal age, education, wealth index, watching television, and contraceptive use were factors associated with the outcome among Bangladeshi women (14). Another study from Malawi evaluated individual and community-level factors of overweight and obesity (15). The results indicated that women in the age group 15–19 years and from the poorest households reduced the odds of overweight and obesity. At the community level, women from the urban areas, with a lower percentage of media exposure, and wealthier communities were the factors associated with increased risk of overweight and obesity (15).

In Africa, policy initiatives to prevent malnutrition target schools, families, and community settings. Currently, the WHO 25 × 25 and the sustainable development goals (SDG), targets 2.2 and 3.4, are in action to reduce the burden of premature mortality from non-communicable diseases (where overweight and obesity are risk factors) and any form of malnutrition (16, 17). Moreover, the WHO Acceleration Plan to Stop Obesity is designed to stimulate and support multi-sector, country-level actions across the globe. The plan focuses on establishing and implementing a data-driven strategy and tries to change obesity prevalence and trends over time until global targets are met (18). As an input to interventions, timely and comprehensive data is necessary, although there has been insufficient data in most countries (19). Congruent with the need for a data-driven strategy, it is crucial to identify priority intervention areas to successfully end these targets. This study aimed to evaluate factors affecting malnutrition among women of reproductive age in sub-Saharan African countries. Identifying the factors influencing malnutrition will help us prioritize intervention areas so that we can move aggressively.

## Methods

### Study setting and data source

This study used DHS data in sub-Saharan African countries. According to the World Bank (20), there are 48 countries in the region. This study included 33 countries in the region with a dataset from MEASURE DHS with BMI measurements. The following is a list of countries in the region included in the current study. Benin, Burkina Faso, Burundi, Cameroon, Chad, Comoros, Congo, Congo D. Republic, Cote d’Ivoire, Ethiopia, Gabon, Gambia, Ghana, Guinea, Kenya, Lesotho, Liberia, Madagascar, Malawi, Mali, Mozambique, Namibia, Niger, Nigeria, Rwanda, Senegal, Sierra Leone, South Africa, Tanzania, Togo, Uganda, Zambia, and Zimbabwe. The list of SSA countries included in the project along with their respective datasets can be found at https://dhsprogram.com/Countries/. The list of countries, the year of the survey, and the sample size are provided in Table 1.

**Table 1:** The list of countries, the year of the survey, and the sample size of each country in the SSA.

	Country	Year of survey	Sample	Percentage
1	Benin	2018	7,827	3.16
2	Burkina Faso	2021	15,654	6.31
3	Burundi	2017	7,747	3.12
4	Cameroon	2018	6,119	2.47
5	Chad	2015	9,426	3.8
6	Comoros	2012	4,766	1.92
7	Congo	2014	4,927	1.99
8	Cote d’Ivoire	2012	6,635	2.68
9	DR Congo	2021	7,892	3.18
10	Ethiopia	2016	13,505	5.45
11	Gabon	2021	5,557	2.24
12	Gambia	2020	5,350	2.16
13	Ghana	2022	6,919	2.79
14	Guinea	2018	4,782	1.93
15	Kenya	2022	15,377	6.2
16	Liberia	2014	3,726	1.5
17	Lesotho	2020	6,257	2.52
18	Madagascar	2021	8,704	3.51
19	Malawi	2016	7,274	2.93
20	Mali	2018	4,436	1.79
21	Mozambique	2011	11,877	4.79
22	Namibia	2013	4,008	1.62
23	Niger	2012	4,244	1.71
24	Nigeria	2018	12,984	5.24
25	Rwanda	2020	6,792	2.74
26	Senegal	2011	5,126	2.07
27	Sierra Leone	2019	6,898	2.78
28	South Africa	2016	3,222	1.3
29	Tanzania	2022	6,867	2.77
30	Togo	2014	4,326	1.74
31	Uganda	2016	5,272	2.13
32	Zambia	2014	14,510	5.85
33	Zimbabwe	2015	8,905	3.59
	Total		247,911	100

### Sampling method

The DHS sample is representative at the national, urban–rural, and regional levels. The program uses a standard uniform methodology for all participating countries. Generally, a stratified two-stage cluster design was employed. In the first stage, enumeration areas were selected from the recent census. In the second stage, households were selected from an updated list of households. In the first stage, the enumeration areas are selected based on probability proportional to the size of each stratum. A sample of a predetermined number of enumeration areas is selected independently, with the probability proportional to the size measure of each enumeration area. In the selected enumeration areas, a listing of the households was performed. In the second stage, a fixed or a variable number of households is selected from the complete list using systematic sampling. In each selected household, a household questionnaire is administered to identify women aged 15–49 years. Eligible women were interviewed using an individual questionnaire, and their responses were recorded in an IR recode file. The details of the sampling method of DHS are available at https://dhsprogram.com/publications/publication-dhsm4-dhs-questionnaires-and-manuals.cfm.

### Population

The target population of the current study consisted of women of reproductive age (15–49 years) in sub-Saharan African countries. Since BMI is not a good indicator of pregnant and puerperal women, these groups were excluded from the analysis. Additionally, some women remained unmeasured during the data collection period and were therefore excluded from the analysis. We used a weighted sample of 247,911 reproductive-age women to determine the associated factors. The sample size for each country is provided in Table 1.

### Study variables

We used the Individual Recode (IR) file of all women aged 15–49 years who were neither pregnant nor in the puerperal period. Our outcome variable from the IR file was BMI (V445), classified according to the WHO category. BMI is calculated by dividing an individual’s weight in kilograms by an individual’s squared height in meters. The variable was classified into categories as follows: “underweight” (BMI < 18.5), “normal” (18.5 ≤ BMI < 25), overweight (25 ≤ BMI < 30), and “obesity” (BMI ≥ 30) (21). Based on DHS methods, women whose weight and height were not measured or calculated BMI below 12.0 or above 60.0 were excluded.

The selection of independent variables was based on the previous literature and the availability of these variables in the DHS dataset. This study’s independent variables were classified as individual or community-level variables. The individual-level variables include women’s age in the 5-year group, highest educational level, wealth index, current breastfeeding status, employment status, contraceptive use, parity, media exposure, and marital status. The definitions of these variables according to the DHS are provided in Table 2. Community-level variables include place of residence (urban vs. rural), SSA regions (south, central, west, and east), and survey year.

**Table 2:** Description of independent variables according to the DHS manual.

Variable	Description
Place of residence	The type of residence where the respondent was interviewed was either urban or rural. Urban areas are classified into large cities (capital cities and cities with over 1 million population), small cities (population over 50,000), and towns (other urban areas), while all rural areas are considered to be countryside.
Age	Age of the women in 5-year groups
Educational level	This standardized variable provides education levels in the following categories: No education, Primary, Secondary, and Higher.
Wealth index	The wealth index is a composite measure of a household’s cumulative living standard. It is calculated using easy-to-collect data on a household’s ownership of selected assets, such as televisions and bicycles, materials used for housing construction, and types of water access and sanitation facilities. Generated through principal components analysis, the wealth index places individual households on a continuous scale of relative wealth. DHS separates all interviewed households into five wealth quintiles to compare the influence of wealth on various population, health, and nutrition indicators.
Breastfeeding	Whether the respondent is currently breastfeeding a child
Contraceptive use	Current contraceptives use either modern methods as “yes” or “no” otherwise.
Marital status	Whether the household member is currently, formerly, or never married (or lived with a partner). Currently married includes married women and women living with a partner, and formerly married includes widowed, divorced, or separated women and women who have lived with a partner but are not now living with a partner.
Respondent’s employment status	Whether the respondent was working during the time of the survey or not

For computational feasibility, we redefined some of the variables found in the original DHS dataset. The SSA countries are classified into three categories based on their geographical location. Eastern SSA include Burundi, Comoros, Ethiopia, Kenya, Lesotho, Madagascar, Malawi, Mozambique, Rwanda, Tanzania, Uganda, Zambia, and Zimbabwe. The Western SSA region countries are Benin, Burkina Faso, Cameroon, Chad, Ivory Coast, Gambia, Ghana, Guinea, Liberia, Mali, Niger, Nigeria, Senegal, Sierra Leone, and Togo. The remaining countries, Congo, DR Congo, Gabon, Namibia, and South Africa, are in the central and southern regions.

Year of the survey: Since the survey year span from 2011 to 2022, we categorized the survey years based on the standard 5-year interval used by the DHS (22). Therefore, survey datasets between 2011 and 2014 are coded as 0, datasets from 2015 to 2019 are coded as 1, and datasets from 2020 onward are coded as 2 and are included as independent variables.

Parity: The total number of children ever born is a continuous variable in the original dataset. We classified these data into nulliparous (having no birth), multiparous (having 1–4 children), and grand multiparous (>4 children). The analysis was then performed (23).

Media exposure: The DHS data include information regarding individuals’ engagement with media, specifically listening to the radio, watching television, and reading newspapers. We combined these three variables into a single category with “Yes” and “No” options. If an individual has exposure to one of these media, regardless of the frequency, we classified them as “Yes” and “No” (24).

### Data analysis

We conducted a descriptive analysis to show the characteristics of the included population based on variables. To this end, we performed cross-tabulation of independent variables with BMI classification to provide a sample overview. The description of the sample population was performed after applying sampling weights. The prevalence of underweight, overweight, and obesity among women was assessed after applying sampling weights for each country. We also pooled the prevalence of underweight, overweight, and obesity using the random effects method for the 33 countries.

The initial plan was to conduct multilevel ordinal regression due to the ordered nature of the outcome variable. However, since the proportional odds assumption was not fulfilled, the analysis was switched to multilevel multinomial analysis. We have employed a multilevel multinomial logistic regression analysis using normal BMI as a reference, which includes both random-effects and fixed-effects models. This statistical approach was performed in Stata version 17.

Four different nested models were fitted, with assumptions checked and bivariable analysis performed using a *p*-value threshold of 0.2. The models included the null model (containing only the outcome variable), model 1 (a model fitted using individual-level variables only), model 2 (a model fitted using community-level variables), and model 3 (fitted using both individual and community-level variables). The final interpretation of the results was based on the best model, which was selected using the log-likelihood and Akaike information criterion. In the random-effects analysis, the Intraclass Correlation Coefficient (ICC) was used to assess the variability of the outcome between clusters/communities.

The model (25) is


logyij−spyij−normal−Xβs+uojs,

Where,

-
$y_{ij}$ is the nutritional status of individual I residing in cluster J.
-
S is the outcome (underweight, obese, and overweight).
-
X is the matrix of independent variables at both the individual and cluster levels.
-
$\beta^{\left(s\right)}$ is the effect size of each independent variable on the probability of women being underweight, obese, and overweight relative to normal.
-
${u_{oj}}^{\left(s\right)}$ is a constant term and cross-level interaction term.

The ICC was calculated as the proportion of the between-cluster variation in the total variation (25):


ICC=VaruojVaruoj+π23,

Where,

-
$Var\left(u_{oj}\right)$ is the community (cluster) level variance.

The variability in the odds of obesity and overweight explained by successive models was calculated by the proportional change in variance (PCV) as follows:


$$PCV=\frac{V_{e}-V_{mi}}{Ve}\text{,}$$

where

-
$V_{e}$ is the variance in obesity and overweight in the null model
-
$V_{mi}$ the variances in the successive models.

Finally, the adjusted odds ratio (AOR) with a 95% confidence interval (CI) was reported, and variables with a *p*-value of <0.05 in the multivariable analysis were declared to be significant predictors of the outcome.

## Results

### Description of maternal characteristics

A total weighted sample of 247,911 reproductive-age women was described, and the exploration of associated factors was included. As indicated in Table 3: Description of women of reproductive age in SSA and distribution of BMI categories in each sub-group of variables based on recent demographic and health survey, 60% of the included population were rural residents. One in five reproductive-age women were in the age group 15–19 years. Three-fourths of the women (74.57%) were not breastfeeding during the survey.

**Table 3:** Description of women of reproductive age in SSA and distribution of BMI categories in each sub-group of variables based on recent demographic and health surveys.

	Underweight	Normal	Overweight	Obese	Overall
Place of residence					
Urban	8,063 (8.18)	52,031(52.77)	22,535(22.85)	15,973(16.20)	39.80
Rural	20,055 (13.45)	101,129(67.81)	19,311(12.95)	8,635(5.79)	60.20
Age					
15–19	10,347(19.08)	38,113 (70.29)	4,270(7.87)	1,496 (2.76)	21.89
20–24	4,739 (11.07)	29,820 (69.63)	5,909(13.80)	2,360(5.51)	17.29
25–29	3,640(9.09)	25,358(63.29)	7,397(18.46)	3,668(9.16)	16.17
30–34	2,795(8.02)	19,920(57.12)	7,459(21.39)	4,697(13.47)	14.08
35–39	2,510(8.03)	16,881(54.00)	6,965(22.28)	4,905(15.69)	12.62
40–44	2,147(8.82)	12,748(52.35)	5,422(22.28)	4,035(16.57)	9.83
45–49	1938(9.63)	10,320(51.27)	4,425(21.98)	3,446(17.12)	8.13
Educational level					
No education	10,453(14.53)	47,931(66.62)	9,791(13.61)	3,771(5.24)	29.04
Primary	8,533(11.48)	47,589(64.04)	11,593(15.60)	6,594(8.87)	30.00
Secondary	8,254(9.52)	50,767(58.57)	16,506(19.04)	11,150(12.86)	34.99
Higher	875(5.92)	6,336(46.43)	3,954(26.73)	3,094(20.92)	5.97
Wealth index					
Poorest	7,011(16.81)	29,148(69.89)	3,998(9.58)	1,550(3.72)	16.84
Poorer	6,006(13.45)	30,793(68.94)	5,470(12.25)	2,397(5.37)	18.03
Middle	5,674(11.78)	31,382(65.13)	7,379(15.32)	3,745(7.77)	19.45
Richer	5,035(9.54)	31,005(58.76)	10,506(19.91)	6,221(11.79)	21.30
Richest	4,391(7.27)	30,832(51.04)	14,493(23.99)	10,695(17.70)	24.38
Currently breastfeeding					
No	20,845(11.33)	102,095(59.42)	32,929(17.90)	20,680(11.24)	74.24
Yes	7,273(11.40)	43,687(68.47)	8,916(13.97)	3,928(6.17)	25.76
Contraceptive use					
No	23,538(13.07)	114,041(63.32)	27,428(15.23)	15,104(8.39)	72.70
Yes	4,580(6.77)	39,119(57.85)	14,418(21.32)	9,503(14.05)	27.30
Marital status					
Never in union	11,692(15.66)	49,209(65.89)	9,232(12.36)	4,550(6.09)	30.15
Currently in union	14,186(9.50)	90,237(60.43)	27,937(18.71)	16,965(11.36)	60.28
Formerly in union	2,240(9.44)	13,713(57.81)	4,677(19.72)	3,092(13.04)	9.57
Respondent’s employment status					
No	15,380(13.91)	70,389(63.67)	15,921(14.40)	8,854(8.01)	44.67
Yes	12,708(9.26)	82,594(60.31)	25,897(18.91)	15,748(11.50)	55.33
Parity					
Nulliparous	11,606(16.55)	46,923(66.91)	8,063(11.50)	3,539(5.05)	28.31
Multipara	10,386(8.68)	70,634(59.06)	23,240(19.43)	15,334(12.82)	48.28
Grand multipara	6,127(10.56)	35,602(61.38)	10,543(18.18)	5,734(9.89)	23.42
Media exposure					
No	11,378(15.67)	50,355(69.33)	7,868(10.83)	3,027(4.17)	29.32
Yes	16,740(9.56)	102,805(58.71)	33,977(19.40)	21,580(12.32)	70.68
African region					
Central and Southern	3,375(11.17)	17,179(56.83)	5,566(18.41)	4,107(13.59)	12.20
East	12,468(11.24)	70,604(63.68)	17,031(15.36)	10,779(9.72)	44.76
West	12,275(11.51)	65,376(61.32)	19,248(18.05)	9,722(9.12)	43.04

As indicated in the table, 15,973(16.20%) urban residents and 8,635(5.79%) rural residents were obese. The highest prevalence of obesity was observed among women aged 45–49 years, with 3,222(17.04%) women affected. We can also observe that higher education is associated with a higher percentage of overweight and obese individuals, with 3,860(28.16%) classified as overweight and 2,787(20.34%) classified as obese. In the same scenario, the wealth index also resulted in a higher percentage of overweight and obese individuals, where the richest quantile leading at 14,122(25.30%) classified as overweight and 9,822(17.60%) classified as obese. Mothers who were breastfeeding during the time of the survey had a lower percentage of overweight and obese individuals, with 9,002(15.24%) classified as overweight and 3,541(5.99%) classified as obese. In contrast, those who were not breastfeeding had higher percentages, with 32,942(19.17%) classified as overweight and 18,217(10.60%) classified as obese (Table 3).

The table describes the population under study. The columns in underweight to obese are expressed as the frequency and percentage (row), while the last column is the percentage of each category of the total population. For example, 72.21% of the population had at least one media exposure.

### Prevalence of underweight

**Figure 1:**
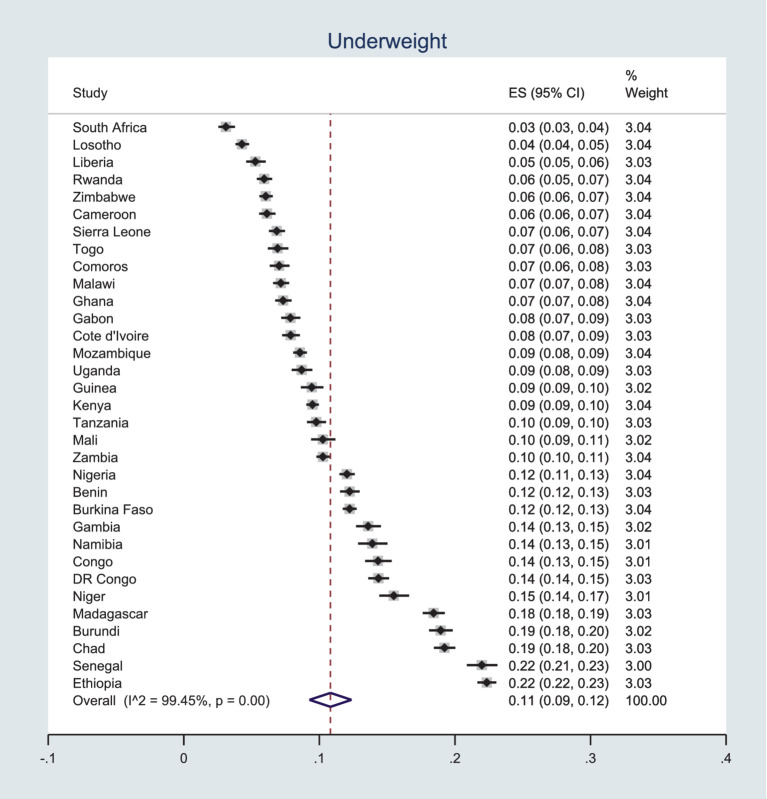
Pooled prevalence of underweight among women in SSA.

The prevalence of underweight in SSA women ranged from 3% in South Africa to 22% in Ethiopia. The pooled prevalence of underweight was 11, 95% CI (9–12%). Figure 1 displayed the country-specific and pooled prevalence of underweight among reproductive-age women in SSA using recent Demographic and Health Surveys.

### Prevalence overweight

**Figure 2:**
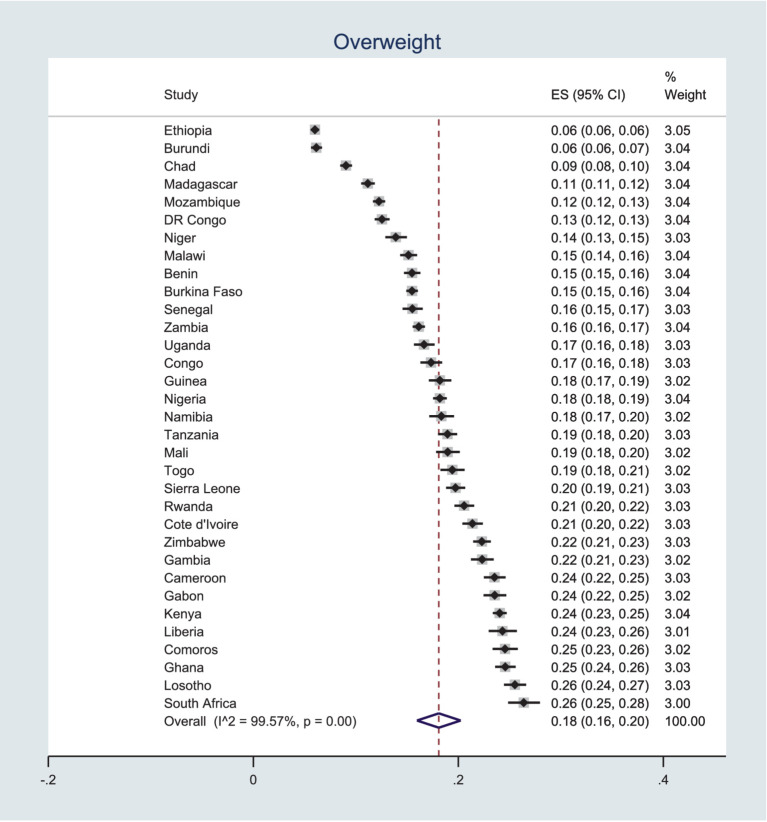
Pooled prevalence of overweight among women in SSA.

Contrary to the results for underweight, Ethiopia has the lowest percentage of overweight women in SSA, with only 6% of reproductive-age women being obese. In stark contrast, South Africa has both the lowest percentage of underweight women and the highest percentage of overweight, at 26%. Using the random effects method to pool country-level percentages, the prevalence of overweight among women in SSA is 18%, with a 95% CI of 16–20%, as indicated in Figure 2.

### Prevalence of obesity

**Figure 3:**
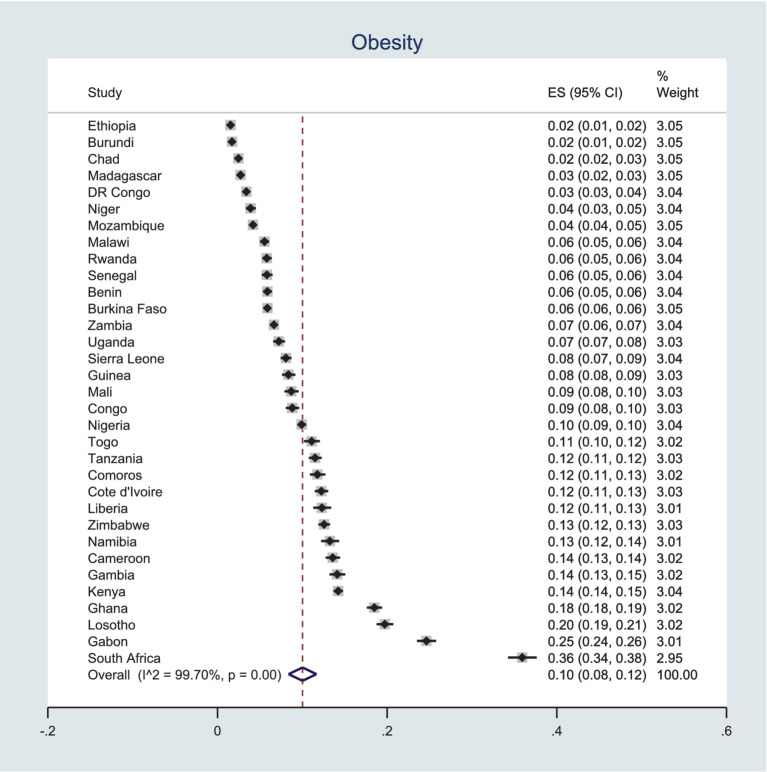
Pooled prevalence of obesity among women in SSA using recent DHSs.

As displayed in Figure 3, the prevalence of obesity in SSA countries ranges from 2 to 36%. Notably, three countries—Lesotho, Gabon, and South Africa—have obesity prevalence rates exceeding 20%. Across the 33 SSA countries included in this analysis, the overall prevalence of obesity is 10%, with a 95% CI of 8–12%.

### Factors associated with underweight, overweight, and obesity

A two-level multinomial logistic regression was used to analyze the association between women’s individual and cluster-level characteristics, as well as nutritional status, as measured by BMI. While the primary objective of this study was to identify factors associated with overweight and obesity, factors influencing underweight were also examined concurrently. According to the null model, 11.10% of the total variance in the odds of malnutrition could be attributed to variations between clusters. In the progression to the final model, the between-cluster variability decreased by 7.92%. Additionally, the Akaike Information Criterion (AIC) and Bayesian Information Criterion (BIC) were lowest in the final model (Model III), making it the preferred model for predicting women’s nutritional status. Factors associated with malnutrition among women of reproductive age in sub-Saharan Africa are detailed in Table 4, where the reference category is women with a normal BMI (not shown in the table).

**Table 4:** Factors associated with nutritional status among reproductive-age women in SSA based on the recent DHS of each country.

Variables	Null	Model I			Model II			Model III		
		Under	Over	Obese	Under	Over	Obese	Under	Over	Obese
Age in years										
15-19(R)										
20–24		0.75(0.71, 0.78)^‡^	1.54(1.47, 1.62)^‡^	1.70(1.58, 1.83)^‡^				0.74(0.71, 0.78)^‡^	1.54(1.46, 1.61)‡	1.66(1.54, 1.78)^‡^
25–29		0.73(0.70, 0.78)^‡^	2.41(2.28, 2.54)^‡^	3.46(3.20, 3.74)^‡^				0.73(0.69, 0.77)^‡^	2.39(2.26, 2.52)^‡^	3.31(3.06, 3.58)^‡^
30–34		0.70(0.66, 0.74)^‡^	3.27(3.09, 3.46)^‡^	6.37(5.89, 6.90)^‡^				0.69(0.65, 0.73)^‡^	3.23(3.05, 3.42)^‡^	6.08(5.62, 6.58)^‡^
35–39		0.73(0.68, 0.77)^‡^	3.87(3.65, 4.10)^‡^	9.04(8.33, 9.80)^‡^				0.71(0.67, 0.76)^‡^	3.80(3.58, 4.03)	8.60(7.92, 9.32)^‡^
40–44		0.79(0.74, 0.85)^‡^	4.31(4.05, 4.59)^‡^	10.99(10.09, 11.96)^‡^				0.78(0.73, 0.84)^‡^	4.23(3.97, 4.51)^‡^	10.50(9.65, 11.43)^‡^
45–49		0.83(0.77, 0.90)^‡^	4.56(4.27, 4.87)^‡^	12.42(11.37, 13.56)^‡^				0.82(0.76, 0.88)^‡^	4.47(4.19, 4.78)^‡^	11.90(10.89, 12.99)^‡^
Educational level										
No education										
Primary		0.80(0.78, 0.83)^‡^	1.22(1.18, 1.26)^‡^	1.75(1.67, 1.83)^‡^				0.76(0.73, 0.79)^‡^	1.30(1.25, 1.35)^‡^	1.77(1.69, 1.85)^‡^
Secondary		0.64(0.61, 0.66)^‡^	1.52(1.47, 1.58)^‡^	2.67(2.55, 2.80)^‡^				0.62(0.60, 0.65)^‡^	1.51(1.45, 1.57)^‡^	2.49(2.37, 2.62)^‡^
Higher		0.70(0.64, 0.76)^‡^	1.66(1.57, 1.76)^‡^	2.90(2.71, 3.10)^‡^				0.68(0.63, 0.74)^‡^	1.66(1.56, 1.76)^‡^	2.77(2.58, 2.97)^‡^
Wealth index										
Poorest (R)										
Poorer		0.82(0.79, 0.85)^‡^	1.20(1.15, 1.26)^‡^	1.15(1.08, 1.22)^‡^				0.83(0.79, 0.86)^‡^	1.19(1.13, 1.24)^‡^	1.13(1.06, 1.21)^‡^
Middle		0.76(0.73, 0.79)^‡^	1.49(1.43, 1.56)^‡^	1.58(1.49, 1.68)^‡^				0.78(0.75, 0.81)^‡^	1.43(1.36, 1.49)^‡^	1.48(1.40, 1.58)^‡^
Richer		0.69(0.67, 0.73)^‡^	1.98(1.89, 2.07)^‡^	2.24(2.11, 2.37)^‡^				0.72(0.69, 0.76)^‡^	1.79(1.71, 1.87)^‡^	1.90(1.78, 2.02)^‡^
Richest		0.67(0.63, 0.70)^‡^	2.56(2.45, 2.68)^‡^	3.28(3.08, 3.48)^‡^				0.69(0.66, 0.73)^‡^	2.20(2.09, 2.31)^‡^	2.48(2.32, 2.65)^‡^
Parity										
Nulli (R)										
Multi		0.81(0.77, 0.85)^‡^	1.23(1.17, 1.29)^‡^	1.53(1.44, 1.63)^‡^				0.82(0.78, 0.87)^‡^	1.19(1.13, 1.25)^‡^	1.46(1.38, 1.56)^‡^
Grand		0.83(0.78, 0.89)^‡^	1.08(1.02, 1.15)^‡^	1.13(1.05, 1.21)^†^				0.84(0.79, 0.90)^‡^	1.07(1.01, 1.14)*	1.10(1.02, 1.18)*
Union										
Never (R)										
Currently		0.83(0.79, 0.87)^‡^	1.16(1.11, 1.21)^‡^	1.11(1.05, 1.17)^‡^				0.82(0.78, 0.86)^‡^	1.22(1.17, 1.28)^‡^	1.21(1.14, 1.28)^‡^
Formerly		0.87(0.82, 0.93)^‡^	1.05(0.99, 1.11)/	0.98(0.91, 1.04)				0.84(0.79, 0.90)^‡^	1.12(1.06, 1.18)^‡^	1.03(0.96, 1.10)
Current working										
No (R)										
Yes		0.70(0.67, 0.72)^‡^	1.01(0.98, 1.04)	0.83(0.81, 0.86)^‡^				0.84(0.82, 0.87)^‡^	0.98(0.95, 1.00)	0.84(0.81, 0.87)^‡^
Currently breastfeeding										
No (R)										
Yes		1.04(1.00, 1.08)*	0.77(0.75, 0.79)^‡^	0.64(0.61, 0.66)^‡^				1.03(0.99, 1.07)	0.78(0.76, 0.81)^‡^	0.65(0.62, 0.68)^‡^
Contraceptive method										
No (R)										
Modern		0.70(0.67, 0.72)^‡^	1.20(1.16, 1.23)^‡^	1.29(1.25, 1.33)^‡^				0.69(0.66, 0.71)^‡^	1.22(1.18, 1.25)^‡^	1.32(1.28, 1.37)^‡^
Media exposure										
No (R)										
Yes		0.81(0.78, 0.83)^‡^	1.37(1.33, 1.41)^‡^	1.74(1.67, 1.82)^‡^				0.80(0.78, 0.83)^‡^	1.32(1.28, 1.36)^‡^	1.67(1.60, 1.75)^‡^
Place of residence										
Urban (R)										
Rural					1.25(1.21, 1.29)^‡^	0.46(0.45, 0.47)^‡^	0.32(0.31, 0.33)^‡^	1.00(0.96, 1.04)	0.69(0.67, 0.72)^‡^	0.58(0.55, 0.60)^‡^
Year of survey										
2011–2014 (R)										
2015–2019					0.90(0.86, 0.95)^‡^	1.29(1.24, 1.34)^‡^	1.09(1.05, 1.14)^‡^	0.80(0.77, 0.83)^‡^	1.23(1.19, 1.28)^‡^	0.99(0.95, 1.04)
2020–2024					0.88(0.84, 0.91)^‡^	1.46(1.41, 1.52)^‡^	1.19(1.14, 1.24)^‡^	0.95(0.91, 0.98)†	1.36(1.31,1.41)^‡^	1.04(0.99, 1.09)
Subregion										
Central and Southern^®^										
East					0.90(0.84, 0.98)^‡^	0.83(0.79, 0.86)^‡^	0.77(0.73, 0.81)^‡^	0.96(0.91, 1.01)	0.73(0.70, 0.77)^‡^	0.68(0.64, 0.72)^‡^
West					0.97(0.92, 1.02)	0.83(0.79, 0.87)^‡^	0.62(0.60, 0.66)^‡^	0.89(0.84, 0.94)^‡^	0.87(0.83, 0.91)^‡^	0.77(0.73, 0.81)^‡^
^‡^*p*-value of ≤0.001, ^†^*p*-value of ≤0.01, *p-value of ≤0.05, a normal BMI is the reference for the dependent variable [not shown]; the “(R)” reference is for the category of independent variable; for all independent variables, the first category is the reference category.										

	Null model	Model 1	Model 2	Model 3
ICC	0.1109948	0.08277383	0.0897144	0.07918918
PCV	NA	0.2771999	0.2106201	0.3111939
Log-likelihood	−262743.2	−241,236	−257796.8	−240069.9
AIC	525494.4	482,606	515631.6	480303.8
BIC	525536.1	483304.2	515829.6	481158.3
Variance	0.4107494	0.2968897	0.3242373	0.2829267

ICC, intra-cluster correlation; PCV, proportional change of variance; AIC, Akaike Information Criteria; BIC, Bayesian Information Criteria.

In Model I, significant variables determining women’s nutritional status included age, highest educational level, wealth index, current breastfeeding status, employment status, contraceptive use, parity, media exposure, and marital status. On the other hand, place of residence, SSA region, and survey year were identified as cluster-level attributes affecting nutritional status.

Model II demonstrated that 8.97% of the variation in nutritional status was accounted for by differences between communities.

### Factors associated with overweight

Women’s age, highest educational level, wealth index, current breastfeeding status, contraceptive use, parity, media exposure, marital status, place of residence, SSA region, and year of the survey were factors significantly associated with overweight in the final model. As age increases, the probability of being in the overweight category increases relative to normal weight. For example, compared to women aged 15–20 years, women in the age group 45–49 years have increased odds of being overweight [AOR = 4.47, 95% CI (4.19–4.78)] and a *p*-value of <0.001 relative to normal weight (reference). Generally, the odds of being overweight increase with age.

The odds of being overweight increase with higher educational levels and wealth indices. Compared to women with no education, those with primary education are more likely to be overweight, with an AOR of 1.30, a 95% CI of 1.25–1.35, and a *p*-value of <0.001 relative to normal weight. Similarly, when compared to women in the poorest wealth quintile, those in the richest category have significantly higher odds of being overweight, with an AOR of 2.20 (CI: 2.09–2.31) and a *p*-value of <0.001. As illustrated in Table 4, there is a clear trend where the odds of being overweight increase progressively with higher wealth quantiles, with the richest individuals displaying higher odds than those who are merely rich.

Parity, current or formerly in the union, contraceptive use, and media exposure were factors that increased the odds of being overweight relative to normal weight. As the number of children ever born by women increased, either multiparity or grand multiparity increased the odds of being overweight compared to nulliparous women. The use of contraceptives and exposure to media increased the odds of being overweight compared with their relative categories of no contraceptive use and no media exposure.

Breastfeeding significantly decreased the odds of being overweight relative to normal weight. Women who were breastfeeding during the survey had lower odds [AOR = 0.78(0.76, 0.81), a *p*-value of <0.001] compared with those not breastfeeding during the same time.

Place of residence, year of survey, and SSA region were community-level factors affecting the odds of being overweight relative to normal weight. Urban areas are the hotspots for overweight and obesity. As shown in Table 4, reproductive-age women residing in rural areas have lower odds of being overweight compared with rural residents. Compared with the latter, recent survey periods increased the odds of being overweight. SSA regions also have differences in the odds of being overweight. Eastern and Western regions had lower odds of being overweight than the Central and Southern regions. The regional effects were significant for both east and west (a *p*-value of <0.001). Table 4 shows factors associated with overweight and obesity among reproductive-age women in sub–Saharan Africa.

### Factors associated with obesity

The factors associated with obesity are similar to those of being overweight. Women’s age, highest educational level, wealth index, current breastfeeding status, contraceptive use, parity, media exposure, marital status, place of residence, and SSA region were factors significantly associated with being overweight in the final model. Compared with women aged 15–19 years, all other categories had higher odds of obesity relative to normal weight. For example, the age groups 20–24 and 25–29 had higher odds of obesity [AOR = 1.66(1.54–1.78) and 3.31(3.06–3.58)], respectively.

In the same scenario with overweight, the odds of obesity are increased with educational level and wealth index. An increase in both variables increased the odds of being obese relative to normal weight. In both cases, the increment is dose-dependent [an increase in educational level and wealth index leads to increased odds correspondingly]. Women with higher education and the richest wealth quantile had the highest odds of obesity compared with no education and the poorest quantile, respectively [AOR = 2.77(2.58–2.97), and 2.48(2.32–2.65), with a *p*-value of <0.001 in both cases].

Multiparity, currently in the union, contraceptive use, and media exposure were factors that increased the odds of being obese relative to normal weight. As the number of children ever born by women increased (multiparity), the odds of being overweight compared to nulliparous women increased. Grand multiparity was not significantly associated with obesity. The use of contraceptives and exposure to media increased the odds of being obese compared with their relative categories of no contraceptive use and no media exposure.

Working and breastfeeding women had lower odds of obesity compared to those with normal weight. Specifically, women who were working had an AOR of 0.84 (95% CI: 0.81–0.87), and those who were breastfeeding had an AOR of 0.65 (95% CI: 0.62–0.68), both with a *p*-value of <0.001 relative to those who were not working or breastfeeding, respectively.

Place of residence and SSA region were community-level factors affecting the odds of obesity relative to normal weight. Rural women had lower odds for obesity than urban women AOR = 0.58(0.55, 0.60), a *p*-value of <0.001. On the other hand, eastern and western countries had lower odds of obesity compared with central and southern SSA regions. Table 4 shows factors associated with overweight and obesity among reproductive-age women in sub–Saharan Africa.

### Factors associated with underweight

It is not a surprise that the variables that are associated with overweight and obesity are associated with being underweight. The association was inverse of the overweight and obesity. For example, as educational status or wealth index increases, the odds of being underweight decrease. This association seems dose-dependent—the higher the educational status or wealth index, the lower the odds of being underweight.

Other variables that negatively correlate with being underweight include an increase in women’s age, parity, marital status, employment during the survey, contraceptive use, and more recent survey years. Specifically, an increase in a woman’s age significantly decreased the odds of being underweight compared to women aged 15–19 years. Similarly, increased parity and being in a union (currently or formerly) also lower the odds of being underweight. The use of contraceptives is associated with a reduced likelihood of being underweight, with an AOR of 0.69 and a CI of 0.66–0.71. Data from more recent survey years show lower odds of being underweight, suggesting a recent decline in the issue. Additionally, women’s employment status influences both underweight and overweight/obesity rates; women who were employed during the survey had lower odds of being underweight, with an AOR of 0.84 and a 95% CI of 0.82–0.87. The factors associated with being underweight are given in Table 4.

## Discussion

Using two-level multinomial analysis, we analyzed data from SSA countries to examine the factors that contributed to both forms of malnutrition. In this study, increases in women’s age, wealth index, not breastfeeding during the survey, contraceptive use, multiparity, being in a union, and residing in urban regions and southern and central Africa increased the odds of higher BMI. These results are witnessed in earlier studies (6, 13, 14).

In this study, higher household wealth was associated with an increased likelihood of overweight and obesity and reduced odds of being underweight. Similar scenarios were found in developing countries previously (26–28). On the contrary, overweight and obesity were more prevalent among populations with lower socioeconomic status in developed countries (29). Studies argued that women of lower socioeconomic status have access to comparatively lower-cost, energy-dense, nutrient-poor foods that contribute to a higher prevalence of obesity in developed countries (30). In the developing world, this situation is not applicable. Rather, many cultural norms that favor fatter body size might contribute to such differences. Women of wealthier households may have the resources and knowledge of a healthy diet, but they also face several socio-cultural barriers that may prevent them from putting those to use and becoming obese (31).

Older age is positively associated with overweight and obesity, while younger reproductive age is linked to underweight. This finding is not strange, as previous studies recorded such results (32, 33). As age increases, the concentration of growth hormone decreases. The decrease in growth hormone, coupled with other hormonal changes, including steroids and decreased physical activity, leads to a decrease in lean mass and an increase in fat mass in the body (34).

Modern contraceptive use is associated with increased odds of being overweight and obese and decreased odds of being underweight. Contraceptives are synthetic steroids. The crucial metabolic effect of steroids is the accumulation of fats in different parts of the body, which increases fatness and obesity (35). Breastfeeding decreases the odds of overweight and obesity. Breastfeeding is important for maternal glucose and lipid metabolism and decreases the maternal risk for metabolic syndrome, which is linked to obesity (36, 37).

Urban women are more likely to be overweight and obese, while rural women are more likely to be underweight. There has been an increase in BMI and the percentage of obese women in urban Africa (12, 38). Although being underweight persists more prevalent than being overweight in rural areas, the ratio of overweight to underweight is decreasing. There is a faster increment in the prevalence of overweight and obesity in rural than in urban Africa (11). Various factors, including food availability and exertion, may contribute to the higher prevalence of underweight in rural Africa. On the other hand, urban areas are more exposed to processed foods, which promote obesity (39).

Parity significantly influenced malnutrition outcomes in this study, consistent with previous findings (40, 41). The link between parity and obesity/overweight is unclear. Evidence suggests that high concentrations of glucose, fatty acids, and amino acids may contribute to weight gain during pregnancy and increase the risk of obesity later (42). Also, physiological changes during pregnancy, including the release of corticotrophin-releasing hormone and activation of the hypothalamic–pituitary–adrenal axis, which leads to increased cortisol concentration, increase the risk of obesity (43). Married women are more likely to be overweight and obese. Consistent findings were reported previously (44, 45). Possible reasons could be that never-married women may care about managing their weight to attract potential partners, married women have family responsibilities, have less time for physical activity, and the dietary pattern is more likely to change (46, 47).

In this study, education was positively associated with overweight and obesity. Previous studies found concurrent (48) and opposite (49) associations in developing and developed countries. The association between overweight/obesity and education is dependent on the country’s level of development, such that inverse associations are more common in more developed countries, and positive associations are more common in less developed countries (50). In developing countries, illiterates are exposed to laborious occupations, and literates have reduced physical exercise due to a sedentary lifestyle (50). Media exposure increases the likelihood of being overweight and obese and lowers the odds of being underweight, as observed in (51, 52). Media use may exert its role in excessive weight gain through incorrect dietary behavior, unbalanced calorie intake, and reduced energy expenditure (52). Regional differences were observed as well.

### Strengths, limitations, and areas of further research

Our analysis used nationally representative DHS data from most SSA countries. It also used hundreds of thousands of participants. This attribute of the study will allow the study to generalize the population under consideration in the region. As a cross-sectional design endeavor, our analysis cannot establish causality. Despite the identification of factors, our analysis disclosed association, not causality. Causal evidence is necessary to resolve the chicken and egg dilemma. The survey year in a few countries is older than 10 years due to the unavailability of recent datasets, and this data may not represent the current state of these countries. While our study provided a comprehensive overview of the SSA region, country-specific evidence is also necessary. Sub-regional and country-level variations require area-specific research.

## Conclusion

In conclusion, factors such as an increase in women’s age, a higher wealth index, not breastfeeding during the survey, contraceptive use, multiparity, being in a union, residing in urban regions, and living in Southern and Central Africa are associated with increased odds of a higher BMI. When designing interventions for overweight and obesity, emphasis should be placed on wealthier, older, reproductive-age women, urban residents, and multiparous women. Conversely, interventions targeting underweight should prioritize the poorest and youngest segments of the population.

## Data Availability

Publicly available datasets were analyzed in this study. This data can be found: https://dhsprogram.com/data/available-datasets.cfm.
